# Efficacy of AS versus SOX regimen as first-line chemotherapy for gastric cancer patients with peritoneal metastasis: a real-world study

**DOI:** 10.1186/s12876-022-02369-9

**Published:** 2022-06-14

**Authors:** Lingyun Zhang, Jiayu Zhang, Yan Wang, Wei Li, Shan Yu, Qian Li, Yiyi Yu, Tianshu Liu, Yuehong Cui

**Affiliations:** 1 Department of Medical Oncology, Zhongshan Hospital, Fudan University, Shanghai, China; 2 Cancer Center, Zhongshan Hospital, Fudan University, Shanghai, China; 3 Department of Oncology, Shanghai Medical College, Fudan University, Shanghai, China

**Keywords:** Gastric cancer, Peritoneal metastasis, First-line, Lauren type, Preoperative chemotherapy

## Abstract

**Background:**

To compare the prognosis of first-line systemic chemotherapy of AS (Albumin-bound paclitaxel and S-1) versus SOX (S-1 and oxaliplatin) regimen in Chinese gastric cancer patients with peritoneal metastasis.

**Methods:**

This was a real-world study of gastric cancer patients with peritoneal metastasis who have been treated with AS or SOX regimen as first-line chemotherapy. Patients were matched by the method of propensity score matching (PSM). The primary and secondary endpoints were overall survival (OS) and progress-free survival (PFS).

**Results:**

A total of 108 gastric cancer patients with peritoneal metastasis were enrolled after PSM analysis. There was no significant difference between AS and SOX regimen based on gender, age, ascites, treatment cycles, gastric cancer resection, received checkpoint inhibitors, and HER-2 expression after PSM analysis. The median OS (14.13 vs. 11.17 months, *p* = 0.0356) and median PFS (10.30 vs. 6.70 months, *p* = 0.0003) of patients who received AS regimen were longer than those treated by SOX regimen as first-line systemic chemotherapy. In sub-group analysis, the median OS and median PFS were longer for patients in AS regimen than SOX regimen in Lauren diffuse type. The occurrence of toxicity between the two groups was shown no significant difference.

**Conclusions:**

The results verified that AS regimen was more effective than SOX chemotherapy in gastric cancer patients with peritoneal metastasis, especially in Lauren diffuse type.

## Introduction

Gastric cancer is reported as the fifth most common cancer in humans and the third leading cause of cancer-related death in the world [1]*.* The standard first-line systemic chemotherapy for advanced gastric cancer is the combination of fluoropyrimidine and platinum, with trastuzumab used in patients with HER-2 positive [2–4]*.* Currently, the most commonly used first-line chemotherapy for gastric cancer patients is S-1 (tegafur, gimeracil, oteracil) or capecitabine combined with cisplatin or oxaliplatin [2, 5, 6], especially the combination of S-1 with oxaliplatin (SOX) [7]. Although these chemotherapy treatments have survival benefits, the median survival time for patients with advanced gastric cancer was only about one-year time*,* and might worsen in those patients with peritoneal metastasis.

As we knew, one of the major causes of the poor prognosis in gastric cancer patients was peritoneal metastasis, accounting for 20 to 40% of all deaths. The incidence of peritoneal metastasis in gastric cancer patients was reported at about 40% [8–10]. The median overall survival (OS) of patients in gastric cancer with peritoneal metastasis was once reported as about 3 to 4 months [11]. Despite the progress of cancer treatment, there was still no standard and effective systemic treatment strategy for these patients with one of the main reasons being the peritoneal-plasma barrier which resisted drug diffusion. Therefore, it is necessary to explore a novel treatment method with high penetrability to lengthen the survival period of gastric cancer patients with peritoneal metastasis.

Albumin-bound paclitaxel (ABX), also known as nab-paclitaxel, has a better metastatic effect on tumor tissue and a higher inhibitory effect than solvent-based paclitaxel. ABX plus ramucirumab which served as second-line chemotherapy showed a slightly longer PFS compared to paclitaxel plus ramucirumab in advanced gastric cancer patients with peritoneal metastasis (5.8 vs 3.5 months, HR 0.66; 95% CI 0.40–1.10, p = 0.109) [12]. The weekly ABX regimen showed longer OS than the paclitaxel regimen (9.9 vs. 8.7 months) of peritoneal metastasis in gastric cancer patients in ABSOLUTE trial [13]. A phase II clinical trial of S-1 combined with ABX (AS regimen) in untreated patients with metastatic gastric cancer showed the median OS was approximately 14 months [14]*.* However, until now there was still no efficacy comparison between AS regimen and the standard therapy SOX regimen in gastric cancer patients with peritoneal metastasis. Thus, the objective of this study was to compare the prognosis of patients who received first-line treatment of AS or SOX regimen in gastric cancer patients with peritoneal metastasis.

## Methods

### Study design and patients

This was a retrospective study of gastric cancer patients with peritoneal metastasis between January 2016 and July 2021 conducted at Zhongshan Hospital of Fudan University. This study was approved by the Ethics Committee of Zhongshan Hospital of Fudan University. The committee removed the individual consent requirement due to the real-world study collecting data from medical records retrospectively. All data were anonymous before data processing.

Inclusion criteria: (1) gastric adenocarcinoma confirmed by histology; (2) all patients were diagnosed with peritoneal metastasis; (3) first-line AS or SOX chemotherapy was used; (4) no synchronous or metachronous cancer; (5) Eastern Cooperative Oncology Group performance status 0 or 1; 6) Trastuzumab was allowed to be used if HER-2 positive.

Diagnosis standards of peritoneal metastasis: (1) CT/MRI/PET-CT scan: omental cake, irregular nodules and thickening of the peritoneum, multiple cord shadows in fat space (peritoneum, omentum, mesentery, and intestinal wall), the density of peritoneal adipose tissue increased, the intestinal wall thickened; (2) clinical signs: board-like rigidity of the abdomen which cannot be attributed to other reason except peritoneal dissemination; (3) laparoscopy examination or laparotomy confirmed by the pathological diagnosis of peritoneal metastasis.

### Data collection

Gender, age, chemotherapy regimen, pathological information, dates of diagnosis and follow-up, dates of initiation and termination of chemotherapy, and dates of progress and death were collected. Adverse events were assessed through the National Cancer Institute-Common Toxicity Criteria version 5.0. All patients underwent routine physical, hematological, and imaging examinations.

### Chemotherapy regimen

All patients received first-line systemic chemotherapy under the guidelines of the National Comprehensive Cancer Network (NCCN) and the Chinese Society of Clinical Oncology (CSCO). The AS regimen included 3-week cycles of ABX (125 mg/m^2^ on day 1, day 8 of each cycle, intravenously) plus S-1. The SOX regimen included 3-week cycles of oxaliplatin (130 mg/m^2^ intravenously on day 1 of each cycle) combined with S-1. The oral doses of S-1 were the same in both groups, with 40 mg (BSA < 1.25 m^2^), 50 mg (1.25 ≤ BSA < 1.50 m^2^), and 60 mg (BSA ≥ 1.50 m^2^) bid on day 1 to 14. Patients were allowed to be maintained with a single drug until tumor progression after the combined AS or SOX treatment. Trastuzumab was allowed to be used in gastric cancer patients with peritoneal metastasis with HER-2 positive. There were no restrictions on second or posterior treatment. The number of cycles was determined by researchers. Treatment continued until unacceptable toxicity, disease progression, patients’ refusal, or the decision by physicians.

### Endpoints

The primary and secondary endpoints were OS and PFS, respectively. OS was defined as the period from the initiation date of treatment to the final follow-up or death for any reason. PFS was defined as the period from the initiation date of treatment to the progression date. The progression was determined as the appearance of new lesions in some cases, the appearance or increase of ascites, or the worsening of clinical findings. The assessment of progression was based on RECIST version 1.1. Adverse event (AEs) were graded according to the Common Terminology Criteria for Adverse Events version 5.0.

### Statistical analysis

Age was divided into two groups at 65. Categorical data were displayed in numbers and percentages and further examined by the chi-square test. OS and PFS were estimated by the Kaplan–Meier method. Propensity score matching (PSM) was carried out through a logistic regression model and nearest neighbor matching algorithm with a ratio of 1:1. PSM accounted for factors of age, gender, ascites, and Lauren type. A difference of less than 10% of the absolute value was considered balanced. Estimates of treatment benefits were calculated as hazard ratios (HR) with 95% confidence intervals (CI). Comparisons in categorical data were evaluated by Fisher’s exact test or chi-square test. SPSS 26.0 (IBM, Armonk, NY, USA) and GraphPad Prism 8 (GraphPad Software, La Jolla, CA) were executed for analysis. *p*-value < 0.05 was considered statistically significant.

## Results

### Characteristics of the patients

**Fig. 1:**
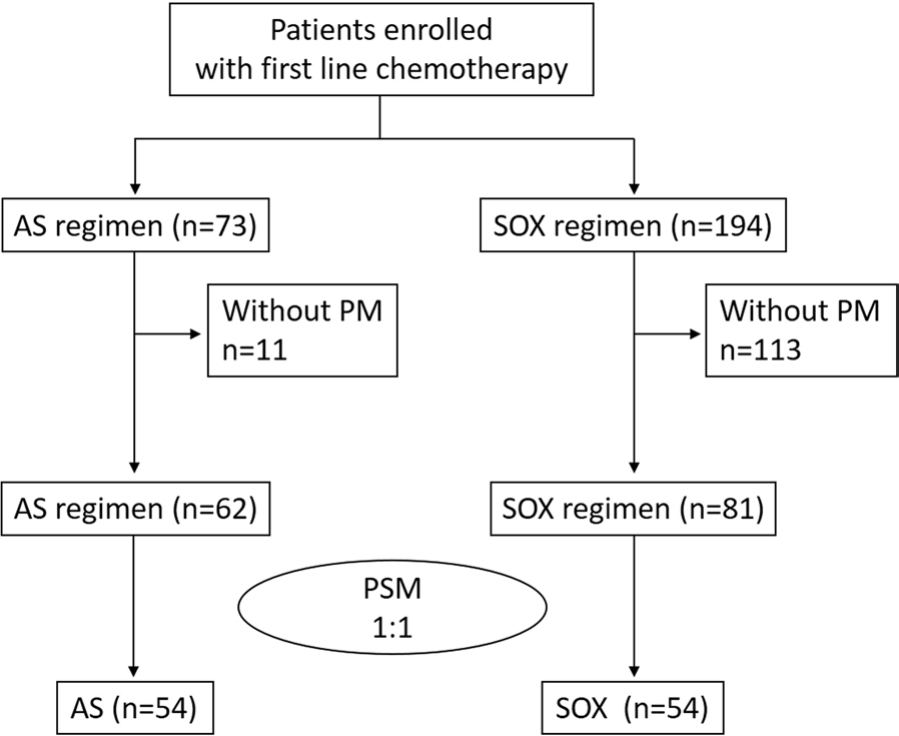
The flowchart of the study. AS, Albumin-bound paclitaxel combined with S-1 regimen, SOX, S-1 combined with oxaliplatin regimen; PM, peritoneal metastases; PSM, propensity score matching

From January 2016 to July 2021, a total of 143 patients met all the inclusion criteria, including 62 cases of AS regimen and 81 cases of SOX regimen. There were 54 cases in each group after PSM analysis. The second or posterior treatment was no restriction. The flowchart of the patients’ enrollment was displayed in Fig. 1. Comprehensive data collection and monitoring were conducted for all patients. The ultimate date of follow-up was July 31, 2021. The baseline features of patients before and after PSM were presented in Table 1. The two groups were all well balanced concerning age, gender, ascites, and Lauren tissue type after PSM analysis. Total of 113 patients died (79.6%; 38 and 75 in the AS and SOX groups, respectively) by the final follow-up day. The median cycles of chemotherapy were both 6 in two groups and the cycle numbers of less than 3 and more than 3 in AS and SOX group was balanced by PSM analysis. To further minimize the effect of other variables, we analyzed the surgery and immune checkpoint inhibitor implement in these patients which were all no significant difference after PSM. Five patients in AS and five patients in SOX group underwent palliative surgery. There were 8 patients in AS group and 5 patients in SOX group treated with a checkpoint inhibitor, including 6 patients treated with PD1 inhibitor and 2 patients treated with MET inhibitor in AS regimen, while 5 patients used PD1 inhibitor and 1 patient used MET inhibitor in SOX regimen after PSM analysis. The common used PD1 inhibitors were nivolumab, pembrolizumab, and toripalimab. There were 5 patients with HER2 positive expression in both groups after PSM, while 4 of them were taken with trastuzumab during first-line treatment in each group. Thus, the two groups were also well balanced in terms of the number of chemotherapy cycles, palliative surgery, HER2 expression, and immune checkpoint inhibitors application.

**Table 1:** Comparison of characteristics before and after propensity score matching (PSM)

Variable	Before PSM		*p*-value	After PSM		*p*-value
	AS (%)	SOX (%)		AS (%)	SOX (%)	
Gender			0.0098			1.0000
Male	29 (46.8)	56 (69.1)		29 (53.7)	29 (53.7)	
Female	33 (53.2)	25 (30.9)		25 (46.8)	25 (46.8)	
Age			0.3440			1.0000
≤ 65	48 (77.4)	56 (69.1)		43 (79.6)	43 (79.6)	
> 65	14 (22.6)	25 (30.9)		11 (20.4)	11 (20.4)	
Ascites			0.2177			1.0000
Without	26 (41.9)	25 (30.9)		21 (38.9)	20 (37.0)	
With	36 (58.1)	56 (69.1)		33 (61.1%)	34 (63.0)	
Lauren type			0.0006			0.0172
Intestinal	4 (6.5)	26 (32.1)	0.3169^a^	4 (7.4)	15 (27.8)	0.5255^b^
Diffuse	42 (67.7)	35 (43.2)		34 (63.0)	24 (44.4)	
Mixed	16 (25.8)	20 (24.7)		16 (29.6)	15 (27.8)	
Cycles			0.0290			0.2318
≤ 3	9 (14.5)	25 (30.9)		8 (14.8)	14 (25.9)	
> 3	53 (85.5)	56 (69.1)		46 (85.2)	40 (74.1)	
Surgery			0.6602			1.0000
Yes	5 (8.1)	5 (6.2)		5 (9.3)	5 (9.3)	
No	57 (91.9)	76 (93.8)		49 (90.7)	49 (90.7)	
Inhibitor			0.4099			0.3750
Yes	8 (12.9)	7 (8.6)		8 (14.8)	5 (9.3)	
No	54 (87.1)	74 (91.4)		46 (85.2)	49 (90.7)	
HER2			0.7631			1.0000
Negative	56 (90.3)	75 (92.6)		49 (90.7)	49 (90.7)	
Positive	6 (9.7)	6 (7.4)		5 (9.3)	5 (9.3)	
Trastuzumab^c^			1.0000			1.0000
No	1 (16.7)	1 (16.7)		1 (20)	1 (20)	
Yes	5 (83.3)	5 (83.3)		4 (80)	4 (80)	
Total	62 (100)	81 (100)		54 (100)	54 (100)	

^a^*p*-value between diffuse and mixed lauren type before PSM

^b^*p*-value between diffuse and mixed lauren type after PSM

^c^trastuzumab in HER2 positive patients; Surgery, palliative surgery during or after first line chemotherapy; Inhibitor, checkpoint inhibitor

### Follow-up

**Fig. 2:**
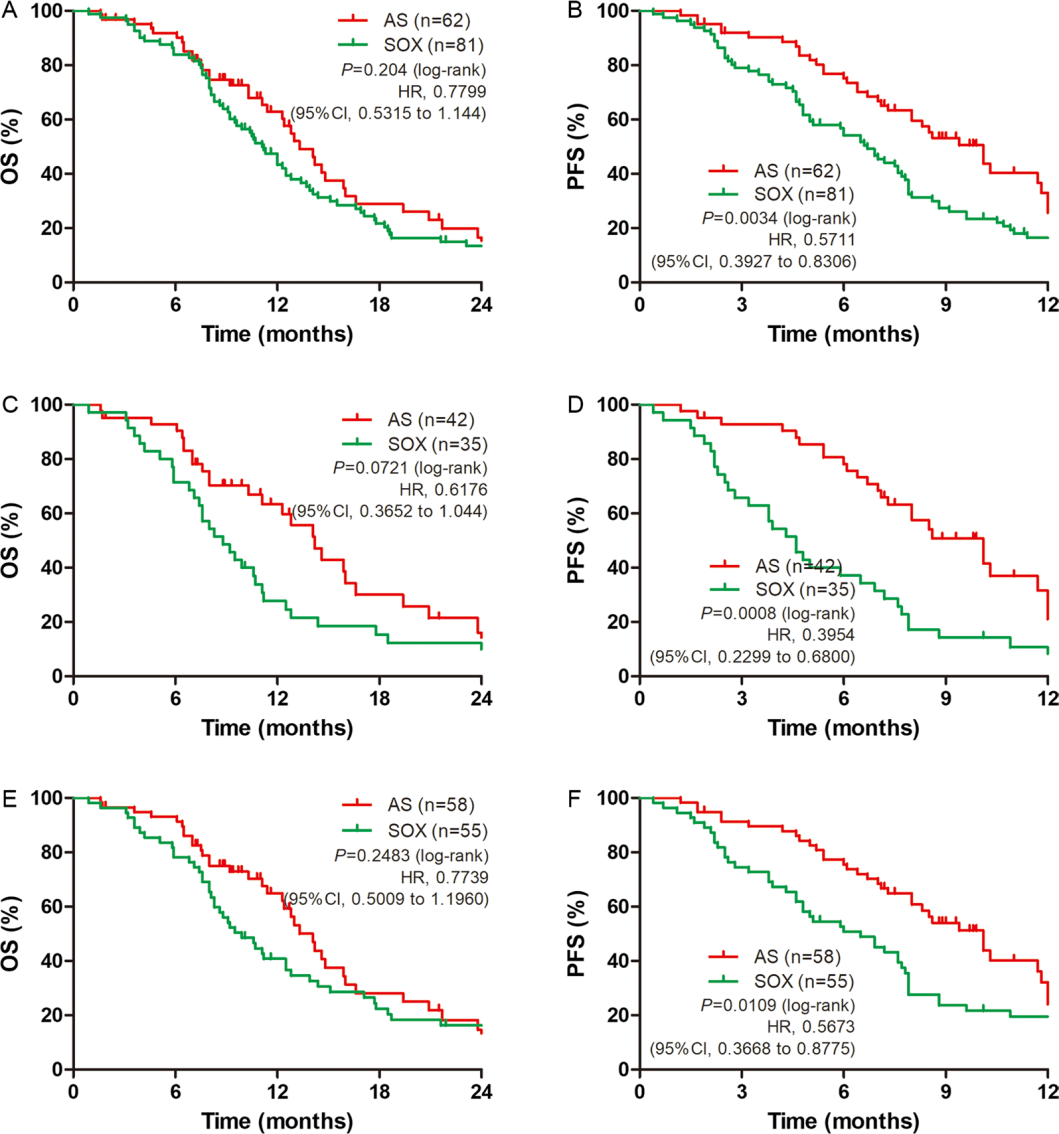
Kaplan–Meier survival curves for overall survival (OS) and progression-free survival (PFS) before propensity score matching (PSM). OS (**A**) and PFS (**B**) analyses for the AS (n = 62) and SOX (n = 81) regimens. OS (**C**) and PFS (**D**) analyses of gastric cancer patients with Lauren non-intestinal type (diffuse and mixed type). OS (**E**) and PFS (**F**) analyses of gastric cancer patients with Lauren diffuse type

The median age of the patients was 58 (range from 19 to 78) years. The median follow-up period was 30.11 months. The median number of chemotherapy cycles was 6 (range from 1 to 9). Before PSM, there was no significant difference between the median OS of patients receiving AS and SOX (HR = 0.7799, 95% CI: 0.5315–1.144) (Fig. 2A; Table 2). However, we discovered a significant difference in median PFS (HR = 0.5711, 95% CI: 0.3927–0.8306) between patients receiving AS and SOX treatment (Fig. 2B). In subgroup analysis, we discovered that there was still no significant difference in diffuse only type and Lauren non-intestinal (diffuse and mixed) types between patients receiving AS and SOX of median OS (Fig. 2C and E). However, a significant difference in median PFS between these sub-groups was discovered (Fig. 2D and F).

**Table 2:** Progress free survival time and overall survival time of patients before and after PSM

Variable	N	OS (months)		PFS (months)	
		Median	95%CI	Median	95%CI
*Before PSM*					
AS	62	13.27	11.35–15.19	10.07	8.40–12.09
SOX	81	11.17	9.61–12.72	6.70	5.51–7.89
AS (non-intestinal)	58	13.27	11.46–15.09	10.07	8.12–12.02
SOX (non-intestinal)	55	9.90	7.71–12.09	6.53	4.31–8.76
AS (diffuse)	42	14.23	11.70–16.77	10.07	7.81–12.33
SOX (diffuse)	35	8.83	6.67–11.00	4.60	3.54–5.66
*After PSM*					
AS	54	14.13	11.96–16.31	10.30	7.06–13.54
SOX	54	11.17	9.09–13.24	6.70	6.08–7.33
AS (non-intestinal)	50	14.23	12.14–16.33	10.30	7.53–13.07
SOX (non-intestinal)	39	9.90	7.30–12.50	6.87	5.26–8.48
AS (diffuse)	34	15.93	13.58–18.28	10.30	7.27–13.33
SOX (diffuse)	24	8.83	6.51–11.15	4.60	2.25–6.95

OS, overall survival time; PFS, progress free survival time; CI, confidence intervals; non-intestinal, including diffuse lauren type and mixed lauren type; PSM, propensity score matching; N, number

**Fig. 3:**
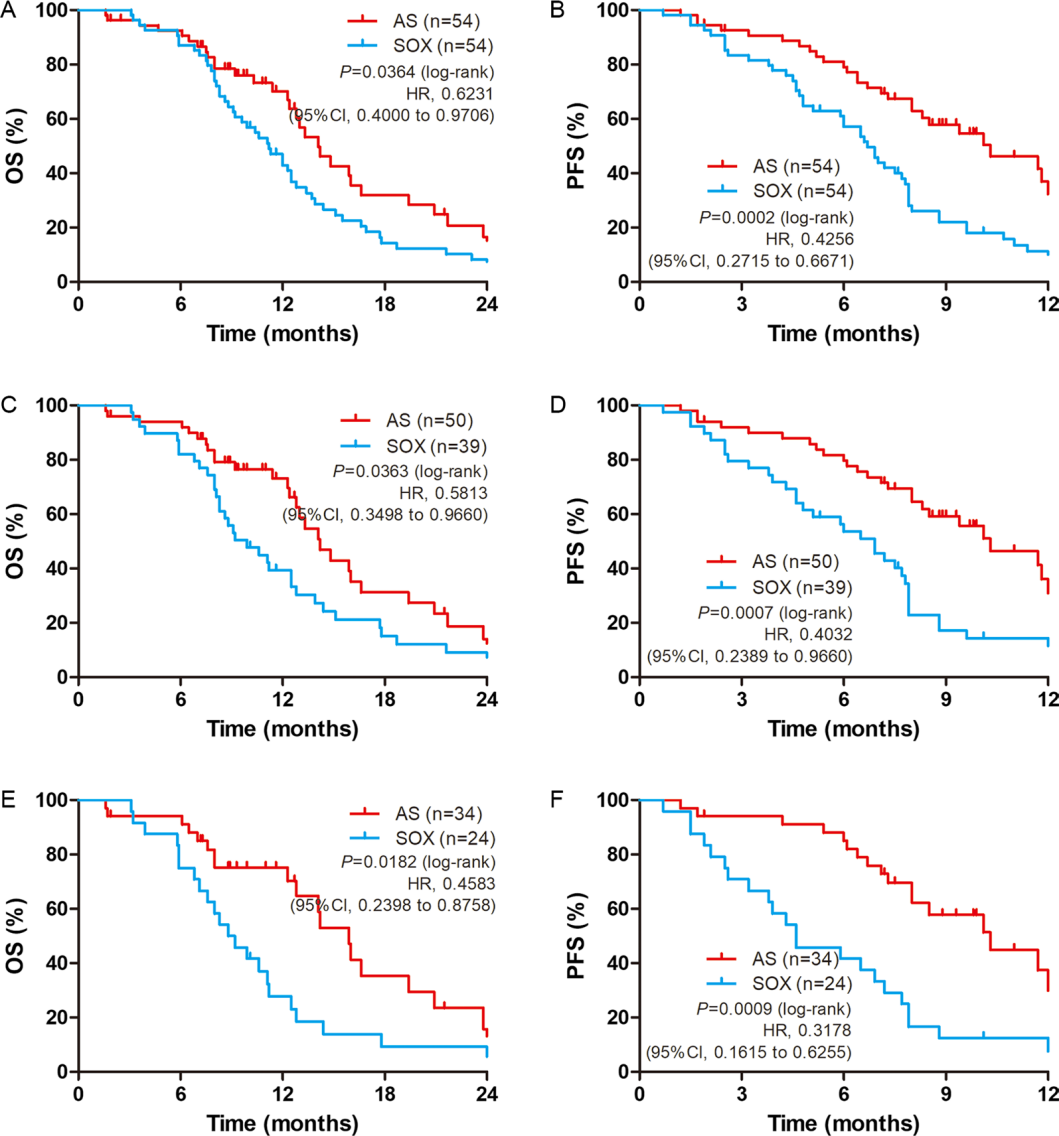
Kaplan–Meier survival curves for OS and PFS after PSM. OS (**A**) and PFS (**B**) analyses for the AS (n = 54) and SOX (n = 54) regimens. OS (**C**) and PFS (**D**) analyses of gastric cancer patients with Lauren non-intestinal type (diffuse and mixed type). OS (**E**) and PFS (**F**) analyses of gastric cancer patients with Lauren diffuse type

After PSM analysis, the median OS was 14.13 and 11.17 months (*p* = 0.0364; HR = 0.6231, 95% CI 0.4000–0.9706) between the AS and SOX groups, while median PFS was 10.30 and 6.70 months (*p* = 0.0002; HR = 0.4256, 95% CI 0.2715–0.6671) respectively (Fig. 3A and B, Table 2). Significant differences were observed in Lauren non-intestinal subgroup (diffuse and mixed type) analysis with median OS (AS 14.23 vs. SOX 9.90 months, *p* = 0.0363; HR = 0.5813, 95% CI 0.3498–0.9660) and median PFS was 10.30 and 6.87 months (*p* = 0.0007; HR = 0.4032, 95% CI 0.2389–0.9660) (Fig. 3C and D). Further in-deep analysis in Lauren mixed type subgroup, we found the median OS (AS 15.93 vs. SOX 8.83 months, *p* = 0.0182; HR = 0.4583, 95% CI: 0.2398–0.8758) and median PFS was 10.30 and 4.60 months (*p* = 0.0009; HR = 0.3178, 95% CI 0.1615–0.0.6225) with significant value (Fig. 3E and Fig. 3F).

### Adverse events

Adverse events of each group were shown in Table 3. The myelosuppression, including leukocytopenia, anemia, and thrombocytopenia was the most frequent hematological adverse event in these two groups. Diarrhea, hepatic dysfunction, vomiting, peripheral neurotoxicity, hand-foot syndrome, and alopecia were also found in each group, but all without significant difference (*p* > 0.05).

**Table 3:** Incidence of adverse events

Event	AS group (n = 62)		SOX group (n = 81)		*p*-Value
	Grade1/2	Grade3/4	Grade1/2	Grade3/4	
Leukocytopenia	3 (4.84%)	3 (4.84%)	2 (2.47%)	N/A	0.0800
Anemia	2 (3.23%)	N/A	1 (1.23%)	N/A	0.4207
Thrombocytopenia	1 (1.61%)	1 (1.61%)	5 (6.17%)	N/A	0.4000
Vomiting	2 (3.23%)	N/A	3 (3.70%)	N/A	0.8816
Diarrhea	2 (3.23%)	N/A	2 (2.47%)	N/A	0.7915
Hepatic dysfunction	1 (1.61%)	N/A	1 (1.23%)	N/A	0.8507
Peripheral neurotoxicity	3 (4.84%)	N/A	2 (2.47%)	N/A	0.5611
Hand-foot syndrome	3 (4.84%)	N/A	2 (2.47%)	N/A	0.5611
Alopecia	3 (4.84%)	N/A	2 (2.47%)	N/A	0.5611

## Discussion

Despite the advances in systemic chemotherapy, the prognosis of gastric cancer patients with peritoneal metastasis remains very poor, even worse than those with other metastatic sites [15]. It was indicated that the peritoneal metastasis rate of gastric cancer patients was about 14% at initial diagnosis, accounting for 20 to 40% of death for gastric cancer which was regarded as the most frequent death cause [11]. To date, peritoneal metastasis is one of the most frequent types of metastasis and recurrence in human gastric cancer. The OS of patients with gastric cancer peritoneal metastasis was about 3–10 months [9, 16, 17]. The first-line systemic strategy for gastric cancer patients with peritoneal metastasis is also the combination of platinum and 5-FU-based regimens [18]. The phase III (SPIRITS) trial proved that cisplatin plus S-1 (CS regimen) was significantly better than S-1 alone for advanced gastric cancer, which was established as the standard first-line therapy in Japan in 2008 [2]. The median OS was longer in patients assigned to CS regimen (13.0 months) than S-1 alone (11.0 months; HR 0.77, 95%CI 0.61–0.98; *p* = 0.04). The median OS of the other commonly used oxaliplatin plus capecitabine (XELOX regimen) was reported as 11.1 months in a phase II study [19]. The median OS of first-line CS and SOX regimen was 13.1 and 14.1 months in the phase III study of patients with advanced gastric cancer respectively [20]_,_ which is similar to the SPIRITS study. In addition, the median OS of paclitaxel with S-1 in advanced gastric cancer patients was displayed as 14.0 months in one randomized phase II study [21]. Here, one thing that needs to emphasize is that the patients enrolled in these trials including advanced gastric cancer patients mostly without peritoneal metastasis. Thus, the median OS of gastric cancer patients with peritoneal metastasis might be worse than that.

Patients with peritoneal disseminated gastric cancer have a low response rate to systemic chemotherapy, mainly due to the existence of the barrier between peritoneal and blood that separates the abdominal cavity from intravenous chemotherapy. Recently, with the emergence of new chemotherapeutic drugs, such as docetaxel and ABX, they have shown good clinical efficacy and controllable toxicity.

It is well known that docetaxel and ABX are very effective for peritoneal metastasis because it has effective transferability to tumor tissues and high antitumor effects for peritoneal metastasis compared with paclitaxel [22–24]. The median OS was 20.0 months with docetaxel and S-1 (DS regimen) and 15.8 months with CS regimen in the phase II HERBIS-3 study [25]. However, the phase III START trial further showed that the DS regimen only improved OS as 12.5 months compared with S-1 alone as 10.8 months (HR of 0.837, 95%CI 0.711–0.985) [26]. In the START trial, the gastric patients with only non-measurable lesions such as peritoneal metastasis showed a better OS of DS regimen than the S-1 alone group, with 17.9 months vs. 12.0 months respectively [26]. A phase I/II study of docetaxel, cisplatin, and S-1 (DCS regimen) enrolled advanced gastric cancer patients with peritoneal metastasis, which showed high anti-tumor efficacy with an OS of 15.5 months but more frequencies grade of 3 or 4 toxicity [16]_._ The HERBIS-3 study reported that the OS of DS regimen was superior to CS regimen in gastric cancer patients with positive peritoneal lavage cytology, with the 2-year OS rates being 70.0% versus 16.7% (HR 0.153, 95% CI 0.037–0.632) [25]. Thus, the docetaxel-based three agents regimen could improve OS of gastric cancer patients with peritoneal metastasis but more toxicity which limits its use in the clinic.

Compared with traditional paclitaxel, ABX has shown significant vasopermeability and tissue penetrability [27]. ABX has many better characteristics than solvent paclitaxel, such as higher plasma clearance and enhanced intratumor delivery, which was encouraged to be used in gastric cancer with peritoneal dissemination [28, 29]. In comparison with traditional paclitaxel, ABX treatment increases the proportion of activated paclitaxel in plasma reported by Gardner et al. [30]. ABX plus ramucirumab was then used in patients with peritoneal metastasis of unresectable advanced or recurrent gastric cancer who have relapsed after first-line therapy [31]. The ABSOLUTE trial showed the weekly ABX regimen had longer OS than the paclitaxel regimen (9.9 vs. 8.7 months) of peritoneal metastasis in gastric cancer patients [13, 32]. Recently, the combination of intraperitoneal paclitaxel and systemic chemotherapy in advanced gastric cancer patients with peritoneal metastasis could enhance the OS to 20.0 months [33]. ABX following intravenous administration was thought to be infiltrated into the peritoneal tumor to the same degree as intraperitoneal injection [34]. Therefore, ABX was a proper systemic agent recommended for the peritoneal metastasis of gastric cancer patients. Our data was manifested that the patients of gastric cancer with peritoneal metastasis who received AS regimen reached a superior median OS (14.13 vs. 11.17 months, *p* = 0.0364) and PFS (10.30 vs. 6.7 months, *p* = 0.0363) than SOX regimen. With the increasing evidence of immune checkpoint inhibitors in gastric cancer, the data of nivolumab plus chemotherapy showed a longer OS and PFS in ATTRACTION-4 and CheckMate 649 trail [35, 36]. However, immune checkpoint inhibitors were rarely used as a first-line treatment in the study. We think combined immunotherapy can prolong the survival time of patients with peritoneal metastasis of gastric cancer, but further research is needed.

Lauren’s classification is the most extensively used classification system of gastric cancer [37–39]. The clinical trial has proved that gastric cancer patients with the diffuse type got a worse prognosis than those with intestinal-type [38]. The report once showed that 46.3% of gastric patients were intestinal type, 32.6% were diffuse type, and 21.1% were mixed type [40]. The OS of gastric cancer patients with diffuse and mixed type was significantly less than those with intestinal-type. Some researchers suggested combining mixed and diffuse gastric cancer into the same category of diffuse-type because the prognosis and survival pattern of the diffuse and mixed survival curves seem to be similar [40]. Our results displayed that about 21.0% of gastric patients with peritoneal metastasis were intestinal type, while 53.8% were diffuse type, and 25.2% were mixed type. Thus, our data indicated that the Lauren diffuse type was the primary type of gastric cancer with peritoneal metastasis. After PSM, the median OS is 14.23 months in AS regimen compared to 9.90 months in the SOX regimen of Lauren diffuse and mixed type (*p* = 0.0363). Further to analyze gastric patients of Lauren diffuse, the OS is 15.93 months in AS regimen compared to 8.83 months in SOX regimen (*p* = 0.0182). The median OS was prolonged from 9.90 months (SOX regimen) to 14.23 months (AS regimen) of diffuse and mixed type in patients with gastric cancer peritoneal metastasis. Meanwhile, the median OS was prolonged by 7.10 months by AS regimen than SOX regimen in Lauren diffuse mixed-type gastric patients (15.93 vs. 8.83 months). Thus, we concluded that patients with gastric cancer with peritoneal metastasis could benefit from AS treatment especially those patients with Lauren diffuse mixed type.

Although there was no difference between our statistics of adverse events in patients treated with AS and SOX, the relatively high incidence of bone marrow suppression was also worthy of attention. We found that leukocytopenia was more common in patients with AS regimen, while the incidence of thrombocytopenia in patients with SOX regimen was higher, which was in line with what we have observed in the clinic. Most AEs were no more than grade 2. There were 3 cases of leukocytopenia and 1 cases of thrombocytopenia with grade ≥ 3 AEs in AS group, while no case with grade ≥ 3 AEs was found in SOX group. The study also had limitations. The main limitation of this study was that it was a nonrandomized retrospective study and the number of cases that we followed up was not enough.

## Conclusions

In this first-line systemic chemotherapy study in Chinese patients of gastric cancer with peritoneal metastasis, we first demonstrated the benefits of AS regimen compared with the SOX regimen. Meanwhile, our data highlighted the evidence that gastric cancer patients of Lauren diffuse-type could get extremely survival time by AS regimen as a first-line strategy. In conclusion, the study indicated that AS regimen was an effective and well-tolerated therapy for the first-line treatment of gastric cancer with peritoneal metastasis, especially in Lauren diffuse type.

## Data Availability

All data generated or analysed during this study are included in this published article.

## Abbreviations

AS: Albumin-bound paclitaxel combined with S-1 regimen
SOX: S-1 combined with oxaliplatin regimen
ABX: Albumin-bound paclitaxel
PSM: Propensity score matching
OS: Overall survival
PFS: Progression-free survival
